# Supraspinatus rupture at the musculotendinous junction in a young woman

**DOI:** 10.1007/s10195-013-0271-x

**Published:** 2013-11-30

**Authors:** Francesco Benazzo, Matteo Marullo, Luigi Pietrobono

**Affiliations:** 1 Clinica Ortopedica e Traumatologica, IRCCS Policlinico S. Matteo, Piazzale Golgi, 27100 Pavia, Italy; 2 U.O.C. Radiodiagnostica, IRCCS Policlinico S. Matteo, Pavia, Italy

## Abstract

The vast majority of rotator cuff tears occur within the tendon or as an avulsion from the greater tuberosity. Supraspinatus injury at the musculotendinous junction is a very uncommon event. We describe a case of supraspinatus rupture at the musculotendinous junction, with successful conservative treatment. It occurred in a 23-year-old woman, the youngest patient with this uncommon type of injury. To our knowledge, this is the first case of rupture of the supraspinatus muscle at the musculotendinous junction in a young woman and the second in a woman.

## Introduction

Rotator cuff tears are very common injuries; ruptures generally occur within the tendon or as an avulsion from the greater tuberosity [1, 2]. Musculotendinous ruptures are common both in the upper and in the lower extremities; ruptures of the rotator cuff through the musculotendinous junction are exceptional [3]. We describe a case of traumatic supraspinatus rupture at the musculotendinous junction in a 23-year-old woman, its clinical presentation, and the successful conservative treatment performed.

## Case report

**Fig. 1:**
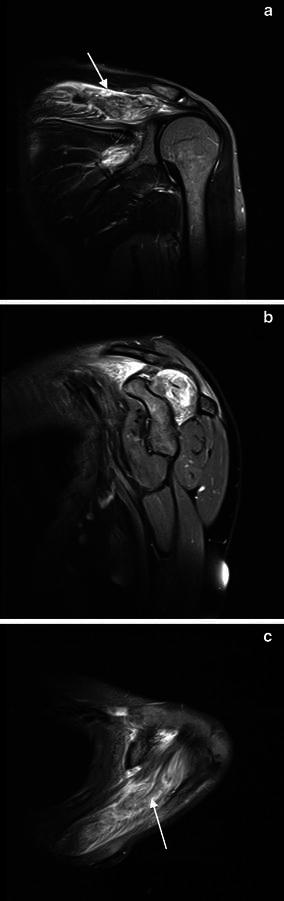
Magnetic resonance imaging performed 1 day after trauma. **a** T1 short-tau inversion-recovery-weighted coronal view shows disruption of the musculotendinous junction of the supraspinatus with hematoma and edema involving the entire muscle belly; the intramuscular septum is inhomogeneous and slackened (*arrow*). **b** Proton density fat-saturated sagittal imaging at the level of the scapular Y view shows a intramuscular hematoma in the posterior portion of the muscle belly and perimuscular edema. **c** The intramuscular septum is clearly slackened on proton-density fat-saturated axial view (*arrow*)

A healthy 23-year-old woman presented to our Emergency Department after a fall on her outstretched left arm, which was forced posterior to her back. Her left, nondominant, shoulder was very painful. On physical examination, there was tenderness over the supraspinatus fossa, no pain on palpation of the scapular body spine or the proximal humerus, and a full passive range of motion (ROM); passive abduction was painful >90°. Active abduction and forward flexion were impossible due to pain. Active internal and external rotation was not impaired. No signs of instability or neurological deficit were found. No fractures or abnormalities were reported on anteroposterior (AP) and axial radiographs. Her pathological anamnesis was uneventful, but she was a smoker. Orally administered analgesics were prescribed, the arm was immobilized in a sling, and a magnetic resonance imaging (MRI) to evaluate the rotator cuff was planned, which showed a complete lesion of the supraspinatus at the musculotendinous junction (Fig. 1).

**Fig. 2:**
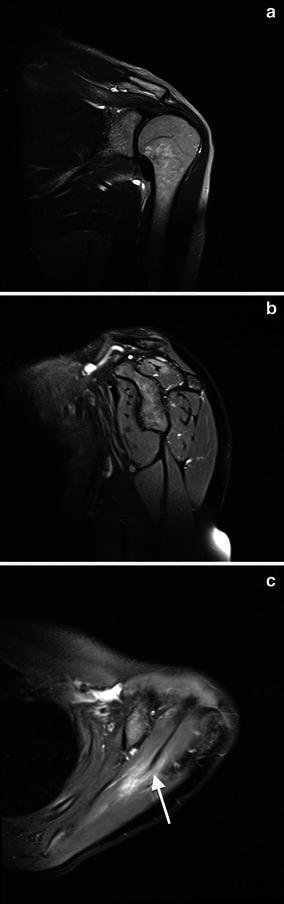
Magnetic resonance imaging performed 60 days after trauma. **a** T1 short-tau inversion recovery coronal and **b** proton-density fat saturated sagittal views show reduction of the intra- and perimuscular edema, but a persistent small intramuscular area of high signal compatible with edema remained in the site of the lesion. **c** Proton-density fat-saturated axial view shows a more homogeneous and stretched intramuscular septum (*arrow*)

The patient was treated conservatively with the arm immobilized in a sling at 45° of abduction and neutral rotation of the shoulder. At day 1, the simple shoulder test (SST) score was 2 (range 0–12) and Constant score was 44. After 25 days, passive elevation, abduction, and circumduction of the shoulder was initiated. After 30 days, assisted active elevation was initiated. At day 40, active abduction and forward elevation to 100° was attained: SST score was 8 and Constant score 57. The patient started eccentric strengthening exercises. At day 60, she had full ROM, including complete abduction; at this time, a repeat MRI showed disappearance of the perimuscular edema and partial reorganization of the muscular architecture (Fig. 2).

**Fig. 3:**
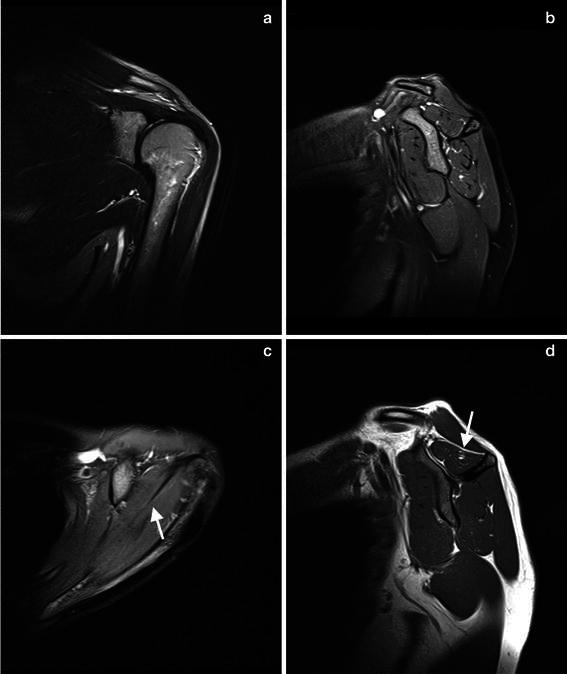
Magnetic resonance imaging performed 29 months after trauma. **a** T1 short-tau inversion-recovery coronal image shows complete disappearance of edema, even though the muscle is hypotrophic. **b** Proton-density fat-saturated sagittal and **c** axial view confirms the reduction of muscle volume and shows a small intramuscular scar in the posterior side of the musculotendinous junction. The intramuscular septum is solid and stretched (*arrow*). **d** Grade 1 fatty degeneration clearly visible on T1 sagittal view (*arrow*)

At day 80, the strength of the supraspinatus was restored to normal, SST score was 12, and Constant score was 100. She was able to perform all activities without limitations. MRI performed 29 months after the trauma showed no edema and a restored intramuscular tendinous septum, but a hypotrophic muscle with a grade 1 fatty infiltration according to Fuchs was visible (Fig. 3). At that time, the patient was healthy, without limitation in her activities, and maintained an SST score of 12 and a Constant score of 100.

## Discussion

To our knowledge, in the peer-reviewed literature there are only six cases of a traumatic rupture of the supraspinatus at the musculotendinous junction [4, 5]. Our patient is the youngest reported with a rupture of the supraspinatus at the musculotendinous junction. Mean age of the six previously reported cases is 40 years [5]; anyway lower than the mean 55 years old found for a supraspinatus tendon tear or the mean 49 years old found for a musculotendinous rupture of the infraspinatus [2, 6]. Musculotendinous injuries are common in the extremities but rare in the rotator cuff; musculotendinous lesions of the supraspinatus are exceptional [5]. Grade 3 injuries are complete rupture of the muscle fibers and generally occur at the musculotendinous junction. The acute phase of such lesions is characterized by widespread intra- and extramuscular edema and hematoma and complete disruption of the muscle; these lesions heal with scar formation and generally muscle weakness [7]. Rupture of the musculotendinous junction may occur at different strain rates according to muscle architecture and elongation.

The main role of the rotator cuff is to stabilize the glenohumeral joint. Consequently, their fibers are designed for force production rather than excursion: they are short and produce near-maximal active tension over a narrow range of sarcomere length. Glenohumeral stability must be provided also with the muscles at rest or in extreme joint position; for this reason, muscle fibers have long resting sarcomere lengths to produce adequate passive tension [8]. The combination of short fibers and long resting sarcomere length make the rotator cuff muscles relatively sensitive to stretch because of the low rate of myofilament overlap [9]. Pennate muscle, such as the infraspinatus, are more susceptible to such injury; for muscles with similar pennation angles but with differing fiber lengths, imposing a given stretch across the muscle will lengthen each sarcomere in a short fiber to a greater extent compared with those in a long fiber [10]. This finding is in agreement with the fact that musculotendinous rupture of the infraspinatus, a pennate muscle, is a recognized injury when rupture of the supraspinatus, a parallel-fibered muscle, is rare; it remains to be understood [6]. 

In our case, the indirect traumatic etiology is clear; the mechanism producing the lesion is debatable. Supraspinatus rupture at the musculotendinous junction could have been caused by a sudden passive stretching of the supraspinatus because of the position of the arm during the fall. When the arm is forced backward and adducted, the supraspinatus fibers are already maximally lengthened and passively stretched, so myofilament overlap is too low to permit muscle contraction. It is well known that reflex muscle contraction is a defense mechanism to prevent or reduce the amount of damage caused by a given injury. Therefore, muscles that are actively contracting, particularly if eccentric, can resist to a greater force before musculotendinous unit failure than can passively stretched muscles. Another explanation for the rupture may be an acute inlet impingement at the acromioclavicular joint during the fall with the arm outstretched and forced backward. The shape of the patient’s acromion, type 2 according to Bigliani, could support this mechanism, but absence of acromioclavicular osteoarthritis could make this mechanism unlikely.

Due to the unique case reported here, we are unable to recommend a treatment strategy for this type of lesion. In our case, conservative treatment showed a satisfactory outcome, leading to complete restoration of supraspinatus function in 80 days and an asymptomatic grade 1 fatty degeneration of the muscle belly on MRI after 29 months. This is in contrast with the previous study in which conservative treatment of full-thickness lesion led to the patient’s dissatisfaction and grade 4 fatty infiltration on MRI follow-up [5].
